# The First Report on the Structure of Polysaccharide Surface Antigens of the Clinical *Klebsiella oxytoca* 0.062 Strain and the Contribution in the Serological Cross-Reactions

**DOI:** 10.3390/ijms26073177

**Published:** 2025-03-29

**Authors:** Agata Palusiak, Anna Turska-Szewczuk

**Affiliations:** 1 Department of Biology of Bacteria, Faculty of Biology and Environmental Protection, University of Lodz, Banacha 12/16, 90-237 Lodz, Poland; 2 Department of Genetics and Microbiology, Institute of Biological Sciences, Maria Curie-Sklodowska University, Akademicka 19, 20-033 Lublin, Poland; anna.turska-szewczuk@mail.umcs.pl

**Keywords:** *Klebsiella oxytoca*, lipopolysaccharide, O polysaccharide, NMR, *Proteus* antisera, cross-reactions, extended-spectrum β-lactamases (ESBL)

## Abstract

*Klebsiella oxytoca* bacilli co-form the human intestinal microbiota, but in favorable conditions, they may also affect immunocompromised individuals, causing urinary tract infections, bacteremia, or antibiotic-associated hemorrhagic colitis. The growing numbers of clinical outbreaks of *K*. *oxytoca* infections make these bacteria an emerging pathogen, which is still masked by the predominant *K*. *pneumoniae* isolates. Thus, it is very important to advance knowledge on *K*. *oxytoca* pathogenicity. This work aims to characterize a urine isolate, *K*. *oxytoca* 0.062, from central Poland, which appears to present a multidrug-resistant and extended-spectrum β-lactamases-positive phenotype. The structural experiments include sugar and methylation analyses, mass spectrometry, and ^1^H and ^13^C Nuclear Magnetic Resonance (NMR) spectroscopy. Additionally, ^1^H,^1^H ROESY, and ^1^H,^13^C HMBC experiments were carried out on the high-molecular-weight O polysaccharide fraction of *K*. *oxytoca* lipopolysaccharides (LPSs). These analyses led to the detection of two polysaccharide antigens: one neutral, containing a linear trisaccharide unit called mannan, and one acidic, which is built up of a branched tetrasaccharide unit containing two mannopyranose (α-Man*p*) residues, one galactopyranose (β-Gal*p*) residue, and one galacturonic acid (α-Gal*p*A) residue. The Gal*p*A residue seems to be a potential minor epitope, recognized by the selected *Proteus* antisera in the serological studies.

## 1. Introduction

*Klebsiella oxytoca* are Gram-negative bacilli that ferment glucose, and like other *Klebsiella* species, they are members of the *Enterobacteriaceae* family. *K*. *oxytoca*, which has been previously called *Bacillus oxytocus* perniciosus, currently forms a heterogenous complex of nine species: *Klebsiella grimontii*, *Klebsiella huaxiensis*, *Klebsiella michiganensis*, *K. oxytoca*, *Klebsiella pasteurii*, *Klebsiella spallanzanii*, and three unnamed novel species [1]. This complex is ubiquitous in the environment and can be found in the human intestines, on the skin, and in the oropharynx. Apart from being human commensals, *K*. *oxytoca* bacilli are also regarded as opportunistic pathogens that may lead to urinary tract infections, bacteremia, or antibiotic-associated hemorrhagic colitis. The latter infection is associated with the production of cytotoxins, including tilivalline, as well as kleboxymycin, which has been characterized in a few representatives of the *K. oxytoca* complex [1,2,3]. Among the other virulence factors found in the *K*. *oxytoca* complex, fimbriae and capsular polysaccharides can be mentioned. A few K types, including K6, K9, K15, K21, K23, K26, K29, K31, K41, K43, K47, K55, K61, K66, K68, K70, K74, and K79, have been identified in the *K. oxytoca* complex [1]. The genome analysis indicated that *K*. *oxytoca* shares some virulence genes with *K*. *pneumoniae* (e.g., *fyuA* and *ybtAPTQ* genes of the yersiniabactin gene cluster, *mrkAB* encoding mannose-resistant fimbriae) [4]. *K*. *oxytoca*, as Gram-negative bacilli, also produce lipopolysaccharides (LPSs) typically built of three regions: lipid A, core region, and O-polysaccharide (an O-antigen). However, there is extremely little information on *K*. *oxytoca* virulence factors, including LPSs [1]. When comparing the structures of the LPS, oligo-, and polysaccharide regions of *K. pneumoniae* to the corresponding structures of *Proteus* spp. LPSs, which is another representative of human commensals from Enterobacterales, some common components were identified. For example, the inner core regions of *Proteus mirabilis* and *K. pneumoniae* LPSs are structurally very similar [5,6]. Additionally, a similar OPS fragment, α-Rha-(1→3)-β-D-GlcNAc(1-, was found in the *K. pneumoniae* O12 OPS and in the O-antigens of *P. mirabilis* O49 and O75, as well as in O51, where α-Rha is 2-O-acetylated [5,7,8]. Such similarity may have resulted in the cross-reactivity of many *Proteus* spp. antisera and a few *Klebsiella* spp. LPSs [9,10]. The *Proteus* spp. OPSs are more structurally heterogenous (over 86 serotypes) than *K*. *pneumoniae* O-antigens (12 O serotypes: O1, O2a, O2afg, O2aeh, O2ac, O3, O4, O5, O7, O8, O12 and O13, and two subserotypes: O3a, O3b) [11,12].

*K*. *oxytoca* has emerged as an important opportunistic pathogen, following *K*. *pneumoniae*, which is the most commonly isolated species. Recent studies have also highlighted the importance of *K*. *oxytoca* as a human commensal by demonstrating its ability to reduce the gut colonization of *K*. *pneumoniae* in mouse models [13].

A great concern is raised by the fact that the isolates from the *K*. *oxytoca* complex carry many genes encoding β-lactamases, including extended-spectrum β-lactamases (ESBLs), such as CTX-M, TEM, and SHV variants, among which the TEM1 β-lactamases are the most common. The adaptation of the *K*. *oxytoca* complex to hospital environments, through its ability to acquire antimicrobial resistance and virulence genes, may be alarming [1]. Thus, it is crucial to expand knowledge on *K*. *oxytoca* features, including antibiotic resistance and pathogenicity, as well as its similarities to other Enterobacterales. This work comprises the results of antibiotic susceptibility testing, beta-lactamases assays, and serological studies on the urine isolate *K*. *oxytoca* 0.062, and provides the structural data on its polysaccharide antigens, such as the LPS O-antigen, which is reported for the first time in this species.

## 2. Results

### 2.1. Characterization of K. oxytoca 0.062 Strain

#### 2.1.1. The String Test and Maneval’s Staining

**Figure 1:**
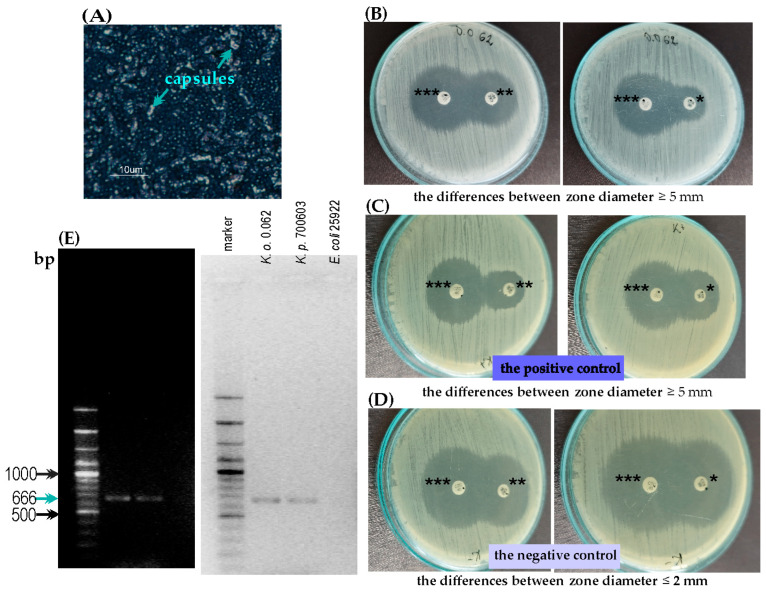
Visualization of *K*. *oxytoca* 0.062 cells using a phase-contrast microscope after staining with Maneval’s method. The capsules are visible as an unstained sphere (marked with the blue arrows) around the stained cells (**A**). The results of combination disk test (CDT) with cefotaxime (CTX) * and ceftazidime (CAZ) **, either alone or in combination with clavulanic acid (CZC or CTC) *** for *K*. *oxytoca* 0.062 strain (**B**), *K*. *pneumoniae* ATCC 700603 (ESBL-positive) (**C**), and *E*. *coli* ATCC 25922 (ESBL- negative) (**D**). The ≥5 mm increase in zone diameter with antibiotic + clavulanic acid, compared to antibiotic alone, indicates the ESBL-positive phenotype. The visualization of the Polymerase Chain Reaction (PCR) product (666 bp) after electrophoresis in 1% agarose at 70 V for 1 h, stained with ethidium bromide (**E**)—in negative and inverted mode—is shown. Lane 1 shows a 100–3000-bp DNA ladder (BLIRT DNA-GDAŃSK).

In the string test, the *K*. *oxytoca* 0.062 did not generate a string effect and did not present a mucoid character of the single colony (it was not viscous enough for a positive result, characterized by a viscous string measuring >5 mm in length). The Maneval’s staining method enabled a detection of thin capsules around the bacterial cells, which was clearly observed in the microscopic preparation by using a phase contrast microscope (Figure 1A). The capsules are visible as unstained spheres (marked with the blue arrows) around the stained cells.

#### 2.1.2. The Antibiotic Susceptibility Pattern and ESBL Detection

The MIC (Minimal Inhibitory Concentration) determination of antibiotics enabled us to demonstrate the multidrug-resistant phenotype of the *K*. *oxytoca* 0.062 strain, which was resistant to the following: norfloxacin, amoxicillin with clavulanic acid, ampicillin, cefuroxim, cephalothin, and trimethoprim + sulfametoxazole. The MIC values obtained for the last four mentioned products were >512 µg/mL (Table 1).

After applying the combination disk test (CDT), the ESBL-positive phenotype was demonstrated for the tested strain. The difference in the diameter of the inhibition zone around the CTX (cefoxitin) disc and the disc with clavulanic acid was 14 mm, and around the CAZ (ceftazidime) disc, it was 7 mm (Figure 1B). For the positive control, *K*. *pneumoniae* ATCC 700603, the difference was 7 mm and 11 mm, respectively (Figure 1C). For the negative control, *E*. *coli* ATCC 25922, the difference was 1 mm for both antibiotic sets (Figure 1D).

When the DNA of the tested strain was subjected to PCR (Polymerase Chain Reaction) using specific primers for the ESBL gene (*bla*_CTX-M-1_), a 666 bp product specific to this gene was detected in the agarose gel for the tested *K*. *oxytoca* strain and the positive control strain, *K*. *pneumoniae* ATCC 700603, confirming their ESBL-positive phenotype. On the contrary, no product was obtained for the negative control of the ESBL production, *E*. *coli* ATCC 25922 (Figure 1E).

### 2.2. Structural Studies of O-Polysaccharide (OPS)

The polysaccharide fraction in the 12% yield was released from the LPS of *K. oxytoca* strain 0.062 by mild-acid hydrolysis followed by the centrifugation of lipid A and isolated by gel-permeation chromatography on a Sephadex G50 fine column. The GLC-MS of the alditol acetates obtained after the full-acid hydrolysis of the polysaccharide showed the presence of mannose (Man) and galactose (Gal) as the major components, in a peak area ratio of 4.7:1.0. In addition to the sugars mentioned above (Man and Gal), galacturonic acid (GalA) was detected by the GLC of the acetylated methyl glycosides. The determination of the absolute configuration of the monosaccharides by the GLC of acetylated (*S*)-(+)-2-octyl glycosides indicated the presence of D-Man and D-Gal. The D-configuration of GalA was revealed by an analysis of the effects of glycosylation on the chemical shifts of carbon atoms, and by a comparison with published data [14,15,16].

The methylation analysis of the polysaccharide fraction resulted in the identification of 1,5-di-O-acetyl-2,3,4,6-tetra-O-methylhexitol-1-*d* (derived from terminal Man), 1,2,5-tri-O-acetyl-3,4,6-tri-O-methylhexitol-1-*d* (derived from 2-substituted Man), 1,3,5-tri-O-acetyl-2,4,6-tri-O-methylhexitol-1-*d* (derived from 3-substituted Man), and 1,3,5-tri-O-acetyl-2,4,6-tri-O-methylhexitol-1-*d* (derived from 3-substituted Gal), with a peak area ratio of 0.5:3:1.0:0.3, identified by GLC-MS. Since in methylation analysis carboxyl-reduction was not applied prior to hydrolysis, no GalA derivatives were detected. The presence of this residue in the polysaccharide was confirmed by NMR.

**Figure 2:**
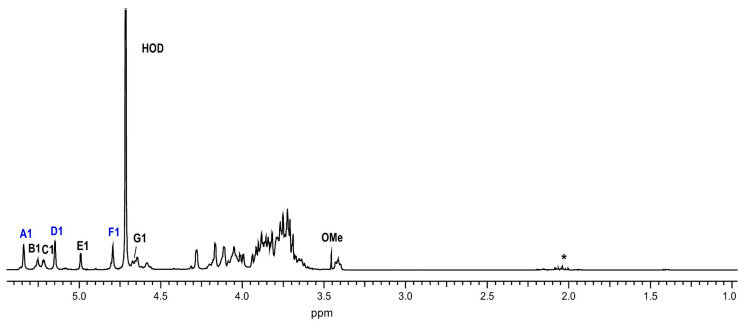
The ^1^H NMR spectrum of the polysaccharide antigens of *K. oxytoca* strain 0.062. The spectrum was recorded at 32 °C in D_2_O at 500 MHz. The capital letters and Arabic numerals refer to the atoms in the sugar residues denoted, as shown in Table 2. The units marked in blue and black indicate the sugar residues of the neutral and acidic polysaccharide, respectively. An asterisk indicates NAc proton signals of the muramic acid as contaminants.

The ^1^H NMR spectrum of the polysaccharide fraction of *K. oxytoca* strain 0.062 (Figure 2) showed seven major signals for anomeric protons at δ 5.34, 5.25, 5.22, 5.15, 4.99, 4.79, and 4.66, with an integral intensity ratio of 1.0:0.8:0.8:1.24:0.7:1.1:1.0, as well as other proton signals in the range of δ 3.41–4.32. The signal at δ 5.15 actually contained two overlapping anomeric proton signals, with the second one having a much lower intensity. Two more signals in the region close to the anomeric proton resonances at δ 4.58–4.64 were assigned to H-4 and H-5 of GalA (see below). In turn, the signal with a lower intensity at δ 3.46 indicated one OMe group.

The ^1^H and ^13^C NMR spectra of the polysaccharide fraction were assigned using two-dimensional homonuclear (^1^H,^1^H DQF-COSY, TOCSY, and ROESY) and heteronuclear (^1^H,^13^C HSQC, ^1^H,^13^C H2BC, and ^1^H,^13^C HMBC) NMR experiments. The ^1^H and ^13^C NMR chemical shifts are collected in Table 2.

**Figure 3:**
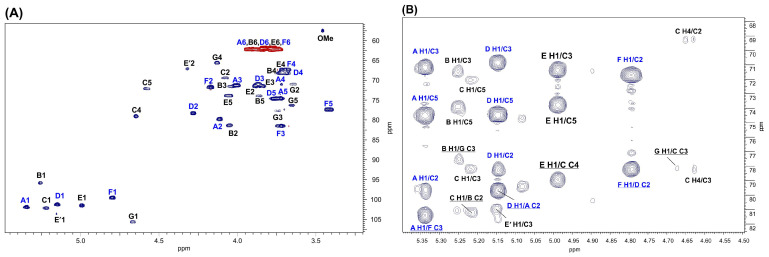
Parts of the ^1^H,^13^C HSQC (**A**) and ^1^H,^13^C HMBC spectra (**B**) of the polysaccharide antigens of *K. oxytoca* strain 0.062. The capital letters and Arabic numerals refer to proton/carbon pairs in the sugar residues denoted, as shown in Table 2. The signal of an α-Gal*p*A H-3/C-3 pair (C3) was visible after increasing the intensity of the spectrum signals (**A**). The long-range interresidue correlations for anomeric protons important for establishing the sugar sequence in the repeating units of the polysaccharides are underlined (**B**). The units marked in blue and black indicate the sugar residues of the neutral and acidic polysaccharide, respectively.

^1^H resonances were assigned using the ^1^H,^1^H DQF-COSY, TOCSY, and ROESY experiments, and on the basis of these data, ^13^C resonances were assigned by ^1^H,^13^C HSQC (Figure 3A) and ^1^H,^13^C HMBC spectra (Figure 3B).

In the ^1^H,^13^C HSQC spectrum (Figure 3A), correlation signals of seven major anomeric atoms at δ_H_/δ_C_ 5.34/101.9, 5.25/95.8, 5.22/102.1, 5.15/101.2, 4.99/101.4, 4.79/99.5, and 4.66/105.6, one minor atom at δ_H_/δ_C_ 5.15/103.6, one of the OMe group at δ_H_/δ_C_ 3.46/57.6, several H-6/C-6 groups of hexoses, and sugar-ring signals were found. The absence in the spectrum of signals in the region of δ 83.0–88.0 characteristics of C-4 furanoses demonstrated that all the sugars building the polysaccharides were pyranoses [17].

The ^1^H,^1^H COSY and TOCSY spectra revealed seven major spin systems, labeled **A**–**F**. Five of them, i.e., the **A**, **B**, **D**, **E**, and **F** spin systems, based on the vicinal coupling constant values, were typical of *manno*-pyranose. In the TOCSY spectrum, the correlations for H-1 with H-2, H-3, and H-5 (**A**, **D**, and **E**) and for H-2 with H-1, H-3, H-4, and H-5 (**A**, **D**, **E**) were visible for almost all spin systems. The remaining resonances were assigned from the DQF-COSY and ROESY spectra and heteronuclear experiments. The α- and β-configuration of *manno*pyranoses were inferred from the chemical shifts for C-5 of δ 73.9–74.5 for the **A**, **B**, **D**, and **E** spin systems, contrary to δ 77.7 for the **F** spin system (compared to published data for α- and β-Man*p* at δ ~74.0 and ~77.0, respectively) [14]. The much less intensive spin system, labeled as **E’**, was also assigned to α-Man*p*. The configuration of linkages was confirmed in the ROESY spectrum by intraresidue H-1/H-2 correlations observed for α-*mann*opyranoses and additional H-1/H-3 and H-1/H-5 correlations for β-*manno*pyranose (spin system **F**).

The remaining two spin systems, **C** and **G** (H-1/C-1 cross-peaks at δ 5.22/102.1 and 4.66/105.6, respectively), were assigned to *galacto*-configurated monosaccharides. In the TOCSY spectrum, starting from the H-1 proton signal, the correlations with H-2, H-3, and H-4 were visible for both residues. Additional correlations, i.e., H-1 with H-5 and H-4 with H-1, H-2, H-3, and H-5 were observed for residue **C**. The remaining resonances were assigned from the ROESY and heteronuclear experiments. For the spin system **C**, the correlation of H-5 at δ 4.58 to its carbonyl C-6 at δ 175.1 was observed in the ^1^H,^13^C HMBC experiment; in the absence of H-6 proton signals, there was a distinctive/discriminative value. By including anomeric coupling constant values, the spin systems **C** (^3^*J*_H1,H2_ = 3.5 Hz; ^1^*J*_C1,H1_ = 174 Hz) and **G** (^3^*J*_H1,H2_ = 8 Hz, ^1^*J*_C1,H1_ = 161 Hz) were assigned to α- and β-glycosidically linked monosaccharides, as α-Gal*p*A (spin system **C**), and β-Gal*p* (spin system **G**), respectively.

**Figure 4:**
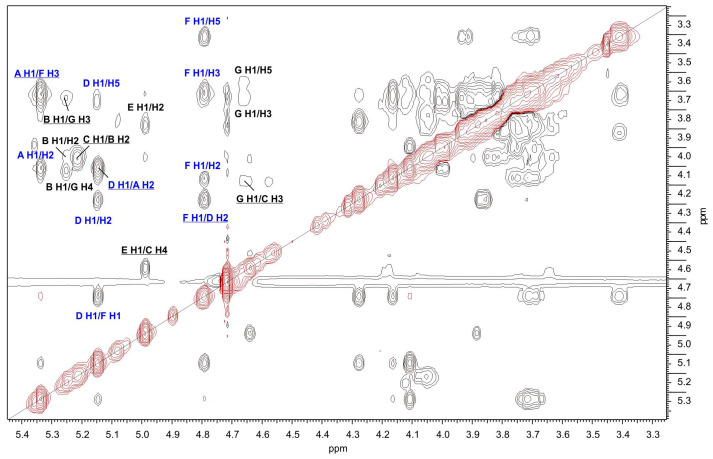
Part of the ^1^H,^1^H ROESY spectrum of the polysaccharide antigens of *K. oxytoca* strain 0.062. The correlations between anomeric protons and protons at the glycosidic linkages are underlined. Some other important H/H correlations are depicted as well. The capital letters and Arabic numerals refer to protons in the sugar residues denoted, as shown in Table 2. The units marked in blue and black indicate the sugar residues of the neutral and acidic polysaccharide, respectively.

The ^1^H,^1^H ROESY spectrum (Figure 4) showed intra-residue cross-peaks: H-1/H-2 for α-Gal*p*A (residue **C**) and H-1/H-3 and H-1/H-5 for β-Gal*p* (residue **G**), which confirmed the anomeric configuration of the sugar residues.

Significant downfield displacements of the carbon atoms for C-2 of residues **A**, **B**, and **D**; C-3 of residues **F** and **G**; and C-3 and C-4 of residue **C** to δ 77.6–81.5 (Table 2), compared with the chemical shifts of the corresponding non-substituted monosaccharides [17], demonstrated the glycosylation pattern of the sugar residues. Chemical shifts of the C-2,3,4,6 carbons of residue **E** were close to those of unsubstituted α-Man*p* [14,18], thus indicating that this residue occupied a terminal position in the polysaccharide. Signal-displacement values observed on the residue **E**’, as compared to unsubstituted α-Man*p* **E**, were due to α-and β-effects of the O-methylation at O-3 (−4.4 ppm, +9.7 ppm, and −0.5 ppm for C-2, C-3 and C-4, respectively). The position of the O-methyl group was confirmed by the heteronuclear correlation between the methyl protons and C-3 of **E’** at δ 3.46/81.0 in the long-range ^1^H,^13^C HMBC spectrum, as described elsewhere [19]. The **E’** residue, which was eventually assigned to α-Man*p*3OMe, occupied the terminal position, similar to residue **E**, indicating that the polysaccharide is non-stoichiometrically methylated (below 15%).

The substitution pattern and sequence of the monosaccharides in the polysaccharide antigens of *K. oxytoca* strain 0.062 was established in the ^1^H,^1^H ROESY and ^1^H,^13^C HMBC experiments. The ROESY spectrum (Figure 4) showed interresidue cross-peaks between monosaccharides, **A**→**F**, **F**→**D**, and **D**→**A**, as well as **C**→**B**, **B**→**G**, **G**→**C**, and **E**→**C**, which, in the absence of additional connecting correlations, indicated the presence of not one but two repeating units building two different polysaccharides. The following strong correlations for the transglycosidic protons were observed: **A** H-1/**F** H-3, **F** H-1/**D** H-2, **D** H-1/**A** H-2 at δ 5.34/3.73, 4.79/4.28, and 5.15/4.12, which indicated the structure of one of the polysaccharides. In turn, the correlation signals for the repeating unit of the second polysaccharide included the following: **C** H-1/**B** H-2, **B** H-1/**G** H-3, **G** H-1/**C** H-3, and **E** H-1/**C** H-4 at δ 5.22/4.05, 5.25/3.74, 4.66/4.18, and 4.99/4.64.

In the ^1^H,^13^C HMBC spectrum (Figure 3B), the correlations between anomeric protons and the transglycosidic carbons confirmed the substitution pattern and sequence of monosaccharides. The following correlations for the first polysaccharide for **A** H-1/**F** C-3, **F** H-1/**D** C-2, **D** H-1/**A** C-2 at δ 5.34/81.5, 4.79/78.2, and 5.15/79.7; and for the second polymer for **C** H-1/**B** C-2, **B** H-1/**G** C-3, **G** H-1/**C** C-3, **E** H-1/**C** C-4 at δ 5.22/81.2, 5.25/77.6, 4.66/78.2 (a weak signal), and 4.99/79.1, were observed.

In summary, the transglycosidic correlations indicate that the first polymer is composed of *manno*pyranoses (α-Man*p* and β-Man*p*), which form a linear trisaccharide repeating unit, and is therefore neutral in character. The second acidic polysaccharide consists of a branched tetrasaccharide unit, and its linear fragment contains α-Gal*p*A, α-Man*p*, and β-Gal*p*, while α-Man*p* occupies a terminal position.

The analysis of the glycosylation effects on the ^13^C chemical shifts confirmed the absolute configuration of Gal*p*A (residue **C**). In the disaccharide fragment **G**-(1→3)-**C**, β-D-Gal*p*-(1→3)-α-Gal*p*A, the large positive α-effect on C-1 of residue **G** (+ 8.2 ppm) and C-3 of residue **C** (+8.0 ppm) indicated the same D-D absolute configuration of the linked monosaccharides. In the disaccharide fragment **C**-(1→2)-**B**, α-Gal*p*A-(1→2)-α-D-Man*p*, the large positive α-effect on C-1 of residue **C** (+9 ppm) and C-2 of residue **B** (+9.5 ppm) and the small negative β-effect on C-1 (−0.8 ppm) and C-3 of residue **B** (−0.2 ppm) indicated the same D-D absolute configuration of the linked monosaccharides [15,20].

**Scheme 1:**
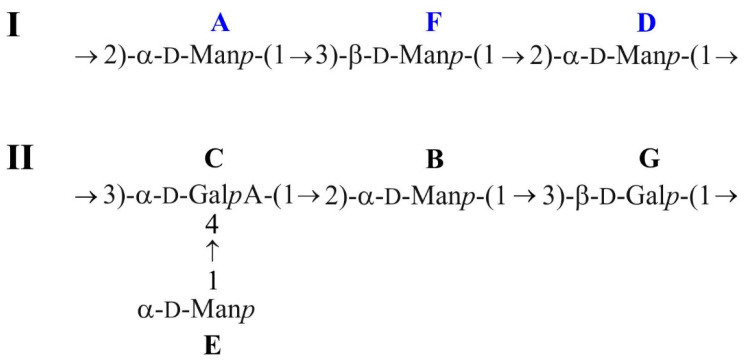
Structure of the polysaccharide antigens of *K. oxytoca* strain 0.062. The neutral polysaccharide (I), an acidic polysaccharide (II).

In conclusion, the data showed that the polysaccharide antigens of *K. oxytoca* strain 0.062 have the structures presented below (Scheme 1):

The presence of α-Man*p*3OMe, which occupies a terminal position, indicates that the polysaccharide is non-stoichiometrically methylated. It is likely that the O-methylation concerns Man*p* in the branched polysaccharide. However, this has not been confirmed due to the lack of unequivocal correlation signals. α-Man*p*3OMe may also be a terminal residue of the neutral polysaccharide, constituting the cap of the O-chain, similarly to what was observed in *K. pneumoniae* O5 serotype [21].

### 2.3. The Electrophoretic Pattern of K. oxytoca 0.062 LPS in Alcian Blue/Silver Staining Method

**Figure 5:**
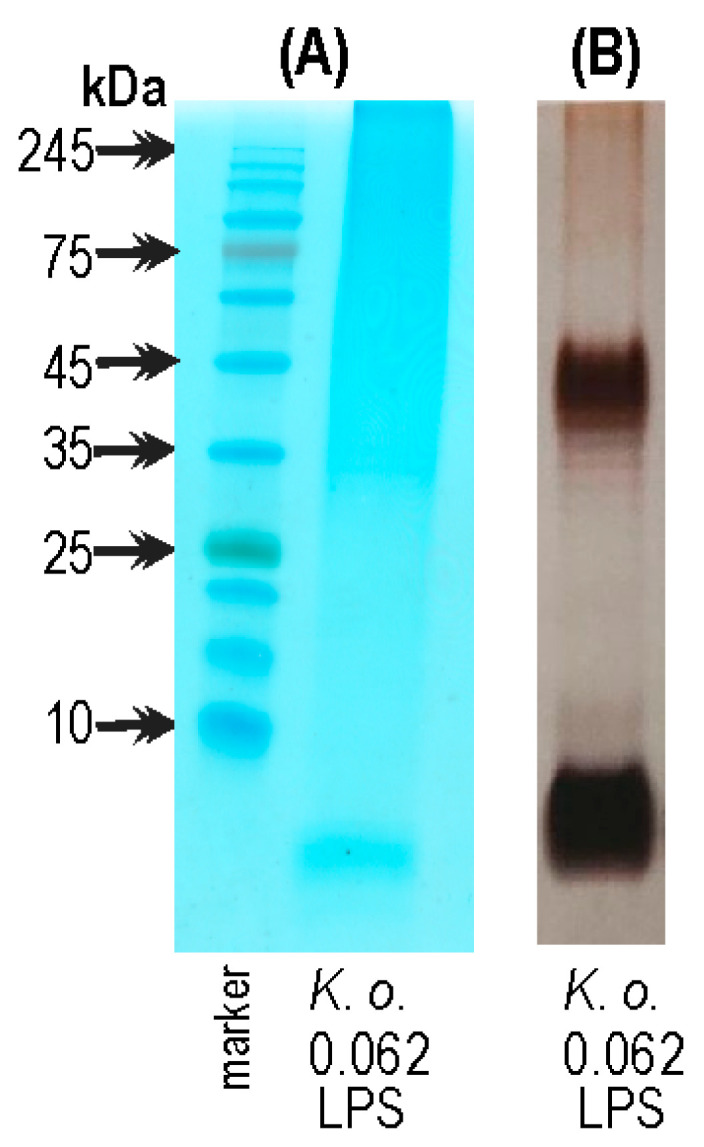
Alcian blue (**A**) and silver staining patterns (**B**) of *K. oxytoca* 0.062 LPS preparation.

Acian blue bound most intensively to high-molecular-weight polysaccharides of *K*. *oxytoca* 0.062 at the level corresponding to the marker proteins of 245-75 kDa (Figure 5A). The silver staining procedure (Figure 5B) additionally revealed two intensively silvered bands of *K*. *oxytoca* 0.062 LPS: the ladder-like pattern typical for high-molecular-weight LPS molecules with O-polysaccharide repeating units (35–45 kDa), and the low-molecular-weight core-lipid A LPS; the latter fraction was only weakly stained with Acian blue (Figure 5A).

### 2.4. Serological Studies

**Figure 6:**
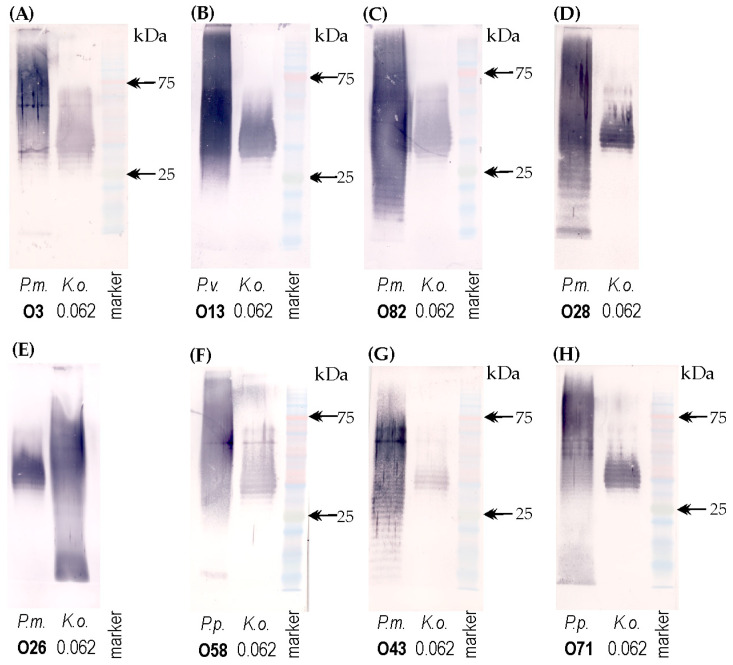
Western blots of *K. oxytoca* 0.062 electrophoretically separated LPSs in cross-reactions with the sera specific to *P*. *mirabilis* O3 strain (**A**), *P*. *vulgaris* O13 (**B**), *P*. *mirabilis* O82 (**C**), *P*. *mirabilis* O28 (**D**), *P*. *mirabilis* O26 (**E**), *P*. *penneri* O58 (**F**), *P*. *mirabilis* O43 (**G**), and *P*. *penneri* O71 (**H**). The *Proteus* spp. LPS homologous to an appropriate serum was applied as a control of the reaction specificity. The solution of each LPS (2 mg/mL) was mixed (1:1) with the sample buffer, boiled for 10 min, centrifuged, and loaded in a stacking gel channels (6 µg/lane). The 3-Color High Prestained Protein Marker (10–245 kDa) DNA GDAŃSK was used to indicate the range of cross-reaction.

After detection by the structural studies, the α-GalA residue in the *K*. *oxytoca* 0.062 polysaccharide antigen encouraged us to check if this acidic component will be recognized by the sera specific to *Proteus* O-serotypes which contain α-Gal*p*A residue/residues (O3, O13, O82, O28, O26, O58, O43, and O71). Thus, these antisera were selected and tested in Western blotting with the *K*. *oxytoca* 0.062 LPS (Figure 6A–H).

The different intensity of the cross-reactions appeared at the level corresponding to high-molecular-weight LPS species (the range between 75 and 25 kDa in the correspondence to prestained protein marker) (Figure 6A–H). The strongest binding patterns were obtained with four *Proteus* antisera specific to O3, O13, O82, and O28 serotypes (Figure 6A–D), whereas the weakest cross-reaction was observed for the tested *K*. *oxytoca* LPS and *P*. *mirabilis* O43 antiserum (Figure 6G). In most cases, the ladder-like pattern was visible, which proves the presence of high-molecular-weight molecules including an O-specific long-chain polysaccharide. The reaction of LPSs homologous to the appropriate *Proteus* antiserum was observed with both low- and high-molecular-weight species, whereas the cross-reactions concerned only the latter species (Figure 6). Therefore, this suggests that the epitopes common to representatives of both genera are located in polysaccharide antigens.

### 2.5. The Electrophoretic Pattern of Cross-Reacting Proteus spp. LPSs in Combined Alcian Blue/Silver Staining Method

**Figure 7:**
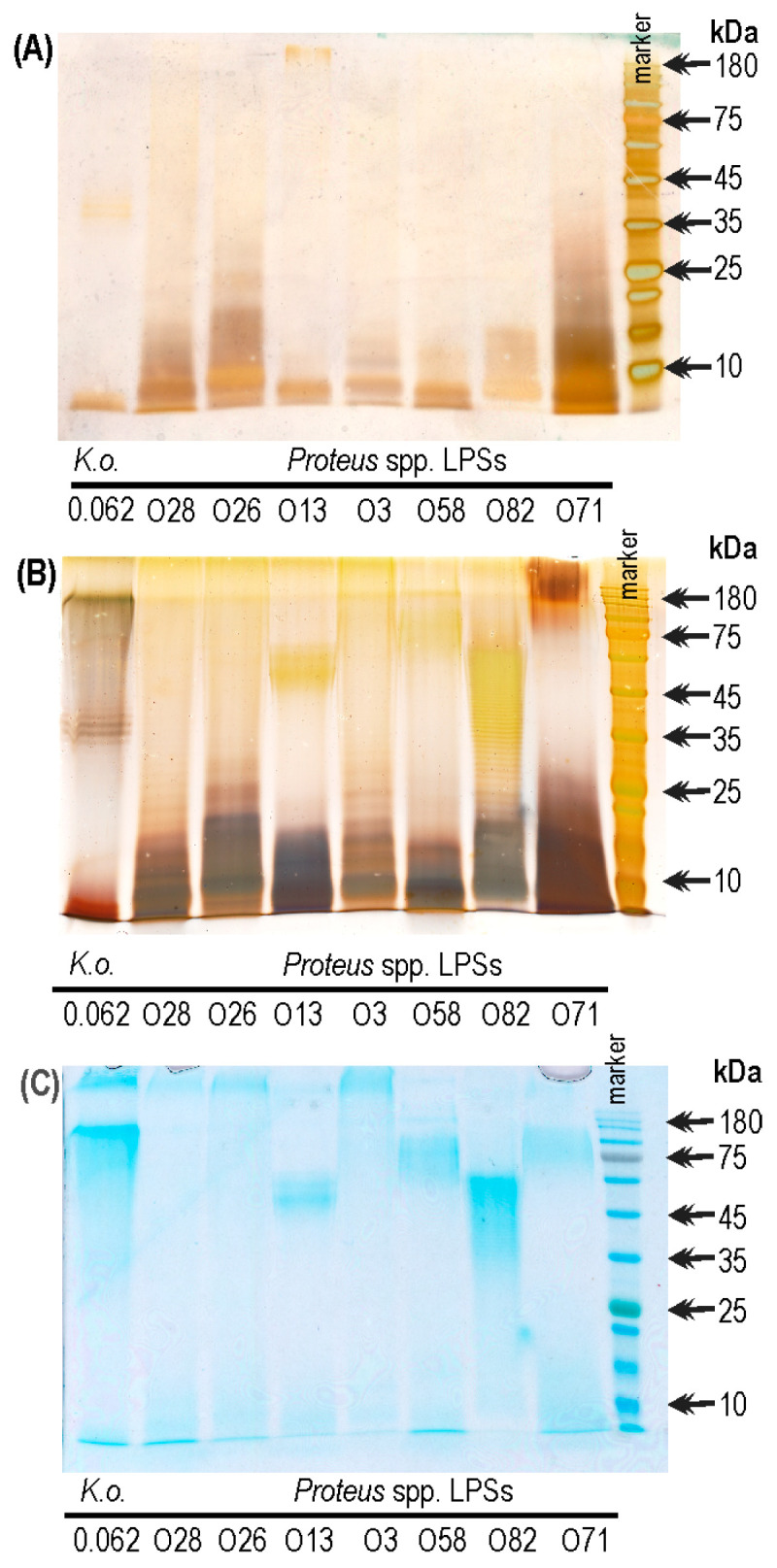
SDS-PAGE and Alcian blue/silver staining patterns of LPS preparations: *K*. *oxytoca* 0.062 and *Proteus* spp. (the sera specific to them cross-reacted in Western blotting with *K*. *oxytoca* LPS) after usage of 0.01% Alcian blue solution (**A**), and 0,1% Alcian blue solution (**B**). Alcian blue staining patterns of *K*. *oxytoca* 0.062 and *Proteus* spp. LPS preparations (**C**).

The *Proteus* spp. LPSs, for which the specific sera were cross-reacted in Western blotting with the *K*. *oxytoca* polysaccharide antigen (Figure 6), were also subjected to SDS-PAGE electrophoresis and visualized by combined Alcian blue/silver staining (Figure 7).

The variability in the intensity of LPS bands is shown in Figure 7. Using the more diluted Alcian blue 0.01% for prestaining the gel resulted in weakly silver-stained high-molecular-weight LPS fractions, among which the most visible ones were obtained for O28 and O26 *Proteus* LPSs (Figure 7A). The most variable patterns were observed for low-molecular-weight fractions corresponding to core-lipid A LPS regions. The *K*. *oxytoca* 0.062 polysaccharide molecules of LPSs were silver-stained only at the level corresponding to the marker proteins of 35–45 kDa (Figure 7A).

Using the more concentrated Alcian blue, 0.1%, revealed more intensive patterns of both high- and low-molecular-weight LPS molecules (corresponding to lipid A-core region), and these latter molecules were the most visible. The most extensive ladder-like patterns (the wider range of reaction) were visible for *P*. *mirabilis* O82 and *K*. *oxytoca* 0.062 LPSs, and their high-molecular-weight molecules were also stained intensively with Alcian blue (Figure 7B,C). Quite intense patterns, characteristic for LPS with long chain O-polysaccharides, were observed for *P. penneri* O71 and *P*. *mirabilis* O13 LPSs; however, they appeared in a narrow range of masses (45–75 kDa, *P*. *mirabilis* O13 and >75 kDa, *P*. *penneri* O71). The remaining LPSs, *P*. *mirabilis* O3, O26, O28, and *P*. *penneri* O58 were silver-stained in a similarly weak way at the level corresponding to their high-molecular-weight molecules (Figure 7B).

## 3. Discussion

*Klebsiella oxytoca* has become an important opportunistic pathogen in humans, capable of causing various types of infections, including hospital-acquired outbreaks. However, its significance is often masked by the more frequently isolated *K. pneumoniae* strains [1]. Limited knowledge about this emerging pathogen, which is of growing importance in clinical outbreaks, highlights the need for further studies on its pathogenicity, virulence factors, and antibiotic resistance. The tested strain, *K. oxytoca* 0.062, is a urine isolate. It is a common source of these bacteria, especially in immunocompromised patients, individuals with underlying genitourinary diseases, and pregnant women. In the latter group, *K. oxytoca* is the second most common cause of UTIs, after *E. coli* [1].

The MIC determination of antibiotics showed that the *K*. *oxytoca* 0.062 strain exhibited the multidrug-resistant phenotype, which also appeared to be an ESBL producer, as revealed by the combination disk test (CDT) (Table 1; Figure 1B). The difference in the diameter of the inhibition zone around the CTX disc and the disc with clavulanic acid was 7 mm greater than the difference obtained for CAZ/CZC discs. Therefore, the CTX/CTC system appears to be more suitable for detecting the ESBL-positive phenotype among *K*. *oxytoca* strains (Figure 1B). However, it should be noted that according to the EUCAST annotation [22], the usage of cefotaxime in ESBL confirmation tests may result in false positive outcomes for *K*. *oxytoca* strains with the hyperproduction of the chromosomal K1 (OXY like) β lactamases. In these studies, an ESBL-positive phenotype was also confirmed by the PCR results (Figure 1E).

Several reports of MDR *K*. *oxytoca* outbreaks can be found. On the other hand, acquired antimicrobial resistance (AMR) genes, including those encoding ESBL, were detected in only six strains out of 239 clinical isolates collected during 15 months from one Australian hospital. The MDR plasmids detected among the Australian isolates were similar to those described for other *Enterobacteriaceae*, which suggests that KoSC has access to the same plasmid reservoir as KpSC [3]. The tested *K*. *oxytoca* 0.062 strain also exhibited the MDR (resistance to six antibiotics) and ESBL-positive phenotypes (Table 1 and Figure 1B), indicating a risk of spreading resistance genes among *K*. *oxytoca* strains, which are isolated less frequently than *K*. *pneumoniae*. The PCR results enabled the detection of the *bla*_CTX-M-1_ gene product, which confirmed the findings of other authors who indicated the *bla*_CTX-M_ genes as relatively frequently detected among *K. pneumoniae* isolates [23,24,25,26]. Ensor et al., 2007 [27] demonstrated the genetic heterogeneity of the CTX-M-1 group of ESBLs, which comprises 20 genotypes, with CTX-M-1, CTX-M-3, CTX-M-10, and CTX-M-15 being the most commonly detected. Their predominance varies by region; for example, the CTX-M-1 type is dominant in Italy, while CTX-M-3 prevails in Poland [27].

The Maneval’s staining method revealed the presence of capsules around the *K*. *oxytoca* 0.062 strain (Figure 1A); however, they were not as extensive as those reported by Dunstan et al. (2021) for the *K*. *pneumoniae* K2 strain, despite the use of a sucrose-enriched medium that stimulates the capsule production [28]. It should be mentioned that sialic acid present in the K2 serotype contributes to the hypermucoviscous phenotype [29], which was not detected for the *K*. *oxytoca* 0.062 strain in a string test. CPS is essential for *Klebsiella* spp. pathogenicity, since it protects bacteria from abiotic stress, antimicrobial peptides, bacteriophages, and complement activity, and enhances adherence and biofilm formation [28,29,30]. Similar functions in *Klebsiella* spp. pathogenicity have also been demonstrated for OPS [30]. Therefore, it is important to analyze the structure of these virulence factors, especially since such data have so far been limited to *K*. *pneumoniae* strains. Both surface antigens are highly immunogenic and induce the production of specific antibodies. As mentioned earlier, the analysis of the OPS structures from *K*. *pneumoniae* and *Proteus* spp. LPSs led to the indication of a few common components [5,7,8]. Both pathogens are known to cause UTIs of a polymicrobial character and were isolated from a mixed biofilm covering the catheters [31,32]. Thus, identifying common epitopes of their surface antigens is crucial for the development of cross-protective antibodies or the selection of vaccine antigens.

In previous studies, the biomass and LPS, obtained from the *K*. *oxytoca* 0.062, have been strongly cross-reacted in ELISA with six *Proteus* antisera [9]. However, the LPS has been extracted from the bacterial cells using the modified Westphal method, in which the crude LPS was precipitated from the water phase with two volumes of 96% ethanol. Such precipitation is also applied in the CPS isolation procedure [33]. In order to check what polysaccharide epitope may be contribute to the cross-reactions, the LPS was extracted from the precisely washed cells (twice in distilled water, and centrifuged at 5300× *g*, 1 h) of *K*. *oxytoca* 0.062 using a standard water–phenol method and purified with 50% TCA, dialysis, and centrifugation. The LPS was subjected to electrophoresis in SDS-polyacrylamide gel, which was stained by using Alcian blue (Figure 5A) or the silver-staining procedure (Figure 5B). This procedure enabled the detection of the most strongly stained high-molecular-weight polysaccharide fractions at a level corresponding to the marker proteins of 75–245 kDa. The ladder-like pattern of high-molecular-weight LPS with O-repeating units occurred after silver staining in the narrow range of sizes, 35–45 kDa (Figure 5B). Thanks to this method, an intensely stained low-molecular-weight core-lipid A fraction of *K*. *oxytoca* 0.062 LPS was also detected (Figure 5B), which was only weakly stained with Alcian blue (Figure 5A).

The high-molecular-weight O polysaccharide fraction was obtained and chemically studied using sugar and methylation analyses, mass spectrometry, and ^1^H and ^13^C NMR spectroscopy, including ^1^H,^1^H ROESY, and ^1^H,^13^C HMBC experiments. These analyses led to the detection of two polysaccharide structures: one neutral, containing a linear trisaccharide unit, and one acidic, which is built up of a branched tetrasaccharide unit. Analyzing the structures of previously established *K*. *pneumoniae* O-serotypes, it is revealed that a similar mannose-containing polysaccharide structure is presented by the *K*. *pneumoniae* O5 serotype. Another serotype possessing only the Man residues but in different linkage configurations is the O3 serotype, which, opposite to the O5 serotype, occurs in two other subserotypes. The previously reported *K*. *pneumoniae* O5 OPS structure includes one modified mannose residue beyond the repeating unit [21]. O5 and O3 serotypes, together with O1 and O2 serotypes, constitute approximately 75–100% of clinical *K*. *pneumoniae* isolates; nevertheless, the O1 serotype is the most abundant [34]. The structural analysis of the polysaccharide fractions also revealed the presence of a branched tetrasaccharide-repeating unit, which is composed of two α-Man*p* residues, one β-Gal*p* residue, and one α-Gal*p*A residue. The data base search indicated that a similar structure is found in *K*. *pneumoniae* K57 and *E*. *coli* K36 serotypes. However, the results by Parolis et al. (1988) slightly differed from those obtained in the present work [35]. The main difference concerned the non-stoichiometric methylation of the terminal α-D-Man*p*, Man*p*3OMe—E’ residue, (Figure 3, Table 2).

**Figure 8:**
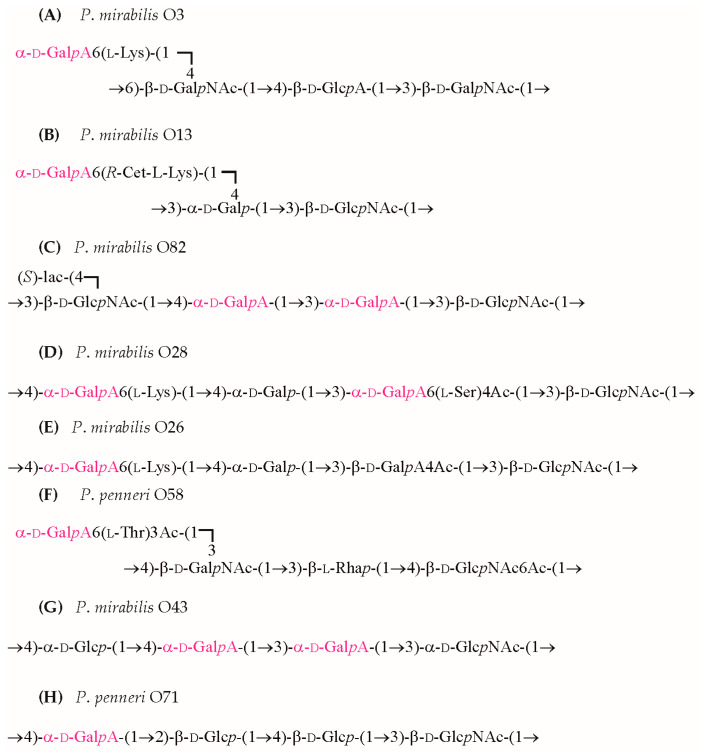
The structures of O-repeating units of *Proteus* spp. LPSs homologous to the sera cross-reacting with *K*. *oxytoca* 0.062 LPS. The components common to all mention LPSs are marked in pink. D-Gal, D-Galactose; D-GalA, D-Galacturonic acid; D-GalNAc, 2-acetamido-2-deoxy-D-galactose (*N*-acetyl-D-galactosamine); D-GlcA, D-glucuronic acid; D-GlcNAc, N-acetyl-D-glucosamine; L-Lys—L-Lysine; L-Rha, L-Rhamnose; L-Ser, L-Serine; L-Thr, L-Threonine; *R*-Cet-L-Lys, N^ε^-[(*R*)-1-Carboxyethyl]-L-lysine; *S*-Lac, (*S*)-Lactic acid [(*S*)-2-hydroxypropanoic acid] [8,37].

Hexuronic acids, including Gal*p*A, are common components of *Proteus* spp. OPSs, in which a form with a free carboxyl group or with a carboxyl group amidated with the α-amino group of amino acids occurs, among which L-lysine is predominant [8]. The amide of Gal*p*A with Lys has been reported to play a crucial role in the epitope specificity of a few *P*. *mirabilis* O-antigens (O3a, O3ab), as well as of the core regions of *P*. *mirabilis* O28, R14/S1959, and *P*. *penneri* O71 LPSs [36]. On the other hand, no Man residues have been found in *Proteus* spp. OPS so far [8]. Considering the above-mentioned facts and the cross-reactivity of different *Proteus* antisera with *K*. *oxytoca* 0.062 LPS (Figure 6), it can be assumed that the α-Gal*p*A residue is recognized by specific antibodies in *Proteus* antisera. Thus, the sera specific to *Proteus* O-serotypes which contain α-Gal*p*A residue/residues, both as a lateral group (O3, O13, O58) or as a part of the main chain of O-repeating unit (O82, O28, O26, O43, and O71), were selected and tested in Western blotting with the *K*. *oxytoca* 0.062 LPS (Figure 6, Figure 7 and Figure 8).

As predicted, the cross-reactions of different intensities were observed at the level corresponding to the 75–25 kDa of the protein marker, and in almost every case, the ladder-like patterns were visible (Figure 6). The α-Gal*p*A residue/residues determine the acidic character of the cross-reacting *Proteus* O-antigens and resulted in Alcian blue staining patterns, among which the ones obtained for *P*. *mirabilis* O82 OPS was the most extensive (Figure 7C). Such an intensity of Alcian blue binding may be associated with the presence of two adjacent α-Gal*p*A residues in *P*. *mirabilis* O82 OPS (Figure 8C) [37]. The results also revealed that 0.1% Alcian blue is more suitable than 0.01% Alcian blue to obtain intense patterns of LPS molecules with strongly charged acidic groups (Figure 7A,B).

Similar patterns as those obtained in Western blotting with *K*. *oxytoca* 0.062 LPS (Figure 6) have been also reported for LPS in *K*. *pneumoniae* O1 whole-cell lysates with polyclonal anti-O2a antibodies. The serum recognized the OPS with shorter chain lengths, for which the ladder-like patterns have also been visible [34].

In polyclonal sera, obtained by the rabbit immunization with the whole-cell suspension, many types of antibodies can be found. In *Proteus* spp. antisera, the O-specific antibodies predominate, among which major- and minor-epitope-specific immunoglobulins can be detected [8]. Considering the cross-reactions observed between *Proteus* antisera and *K*. *oxytoca* 0.062 LPS, which differed from those obtained in each homologous system (Figure 6), it can be concluded that a minor epitope in the *K*. *oxytoca* polysaccharide antigen was recognized by specific antibodies. Although the cross-reactive minor epitopes are usually structurally more complex, a single domain (3-substituted β-D-GlcpNAc residue) has also appeared to be sufficient for a cross-reaction between *P*. *mirabilis* O23 antiserum and the LPSs of *P. mirabilis* O6 [8].

It should be remembered that in *Klebsiella* strains, a CPS surrounds the cells and may hinder the access of the specific antibodies to the O-antigens. Few reports on the ways in which CPS attaches to the cell have been published; the reports indicated the capsule interaction between a lipid moiety and the lipid A-core region of LPS. Fresno et al., 2006 [38] showed that a phenol–water extract contained both GlcA and Man from a K2 capsule as well as the core region sugars, suggesting a non-covalent association of K2-PS and the core LPS. They suggested a role of divalent ions in ionic interaction between the GlcA residue from CPS and the GalA residue from the LPS core region [38].

Our analyses indicated that both *K*. *oxytoca* 0.062 polymers (neutral and acidic) have a similar molecular weight, and their separation is not possible. However, the molecular weight of the capsular polysaccharide should be much higher than that of the O-polysaccharide fraction. The results that support the presence of two polymers in the *K*. *oxytoca* 0.062 O antigen are the serological studies (Figure 6), which show cross-reactions (related to the acidic polymer) at a level corresponding to a lower weight fraction range than that typical for CPS. The K antigens from *E*. *coli* O8, O9, O20, and O101 K serotypes are high-molecular-weight (3 × 10^5^ to 10^6^) heterogeneous acidic polymers. A similar feature is presented by the K antigens of *Klebsiella* [35]. The Alcian staining of the electrophoretically separated *K*. *oxytoca* 0.062 LPS and the biomass of this strain also supports the possibility of the existence of two different OPSs on the cell surface in rather similar amounts (O5 serotype and OPS-like capsule) (Figure S1). Acian blue bound the most intensely to high-molecular-weight polysaccharides at a level corresponding to the marker proteins of 245–75 kDa. Meanwhile, no blue stains can be seen for the biomass remaining after CPS extraction (panel 1) and only small blue patterns were visible for the *K*. *oxytoca* 0.062 biomass treated and untreated with proteinase K (panels 2, 3), and these patterns probably concerned the CPS [Figure S1]. However, the negative string test, very good sedimentation of the bacterial cells during rinsing the cells via centrifugation (the cells with thick capsule do not sediment so well), and thin layer of the capsule detected around the cells after Maneval’s staining (Figure 1A) suggest that capsule may be not essential in the pathogenicity of *K*. *oxytoca* 0.062, which does not exhibit a hypermucoviscous phenotype.

In conclusion, the results of chemical and serological studies suggest O-antigen heterogeneity due to the presence of two different polymers. However, the co-migration of lower-molecular-weight CPS with OPS components cannot be definitely excluded. Therefore, whole-genome sequencing will be performed in the near future on the *K*. *oxytoca* 0.062 strain to elucidate the origin of the acidic polymer.

The structure of *K. oxytoca* 0.062 polysaccharide antigens, presented in this paper for the first time for this species, expands the knowledge of *K. oxytoca*, which shows an alarming tendency to acquire genes encoding antibiotic resistance or virulence factors. In addition, the *K. oxytoca* 0.062 strain produces polysaccharide antigens similar to those previously detected in *K. pneumoniae* strains. This feature, along with “sharing” the genes with *K*. *pneumoniae*, may cause *K*. *oxytoca* to become a major obligate pathogen within the *Enterobacteriaceae* family.

## 4. Materials and Methods

### 4.1. Bacterial Strains

The *K*. *oxytoca* 0.062 strain came from the collection of *Klebsiella* spp. isolates from the urine of patients from the Łódź region, which were gathered in the Department of Biology of Bacteria, University of Lodz (Poland).

The LPSs and the polyclonal rabbit sera specific to the respective *Proteus* O-serogroup (O3, O13, O26, O28, O43, O58, O71, and O82) came from the collection gathered in the Department of Biology of Bacteria.

### 4.2. The Capsule Staining and the String Test

The *K*. *oxytoca* 0.062 strain was cultured from single colonies for 18 h on nutrient bouillon agar. The formation of a string was determined using a bacteriological loop [39]. A positive result is characterized by a viscous string measuring >5 mm in length.

The tested strain was subjected to passage twice through the broth containing saccharose (2g east extract, 250 mg magnesium (VI) sulfate, 1g potassium (VI) sulfate, 2g sodium chloride, 20g saccharose, 2% agar [pH = 6.5]), which enhances CPS production. The capsule was visualized by Maneval’s staining method [40]. The bacterial cells from the 18 h culture in broth containing saccharose were first introduced in to a drop of Congo red solution on a slide. After Congo red dried, the Maneval’s solution (1% acid fuchsin, 5% phenol increases the stain penetration, 30% FeCl_3_, 20% acetic acid) was added to the slide. The acid fuchsin interacts with the bacterial cell and stains the cytoplasm. The capsules were seen on a phase contrast microscope (Nikon ECLIPSE TE2000-s, Tokyo, Japan) as unstained layers surrounding the bright-red-stained bacterial cells.

### 4.3. Antibiotic Susceptibility

The minimum inhibitory concentration (MIC) of ampicillin, cefuroxim, cephalothin, nitrofurantoin, norfloxacin, trimethoprim + sulfametoxazole, and amoxicillin with clavulanic acid was determined using the broth microdilution method in a V-bottom 96-well plate. Each antibiotic with a concentration of 512 µg/mL was added to the first column of the plate and serially diluted in 100 µL of Mueller–Hinton broth to a concentration up to 1 µg/mL. Afterwards, the 18 h bacterial culture (5 × 10^5^ CFU/mL) was added to each well and the plate was incubated for 18 h at 37 °C. The control of bacterial growth does not contain the antibiotic and the antibiotic control does not contain the bacteria culture. The MIC was determined as the lowest antibiotic concentration in which no bacterial growth was observed. All MICs were interpreted according to the EUCAST 2024 guidelines and the appropriate S (susceptible) and R (resistant) phenotype was determined. *E*. *coli* ATCC 25922 was used as a control for antibiotic activity—a susceptible phenotype [41].

### 4.4. Determination of ESBL Phenotype

The ESBL production ability was performed by using a combination disk test (CDT) with cefotaxime (CTX) and ceftazidime (CAZ) alone or in combination with clavulanic acid (CZC or CTC) according to CLSI guidelines [42]. On the MHA plates, which were previously streaked with diluted overnight culture (0.5 MacFarland), ceftazidime (30 μg—BIOMAXIMA, SA, Lublin, Poland) and cefotaxime (30 μg—BIOMAXIMA) disks were placed 20 mm away from the center of the disk with the antibiotic and clavulanic acid (30/10 μg—BIOMAXIMA) [26]. A 0.5 McFarland *K. oxytoca* inoculum was prepared from morphologically similar colonies cultured overnight on a nonselective solid medium. After 18 h of incubation at 37 °C, the ESBL phenotype was interpreted as a positive if the inhibition zone diameter was ≥5 mm larger with clavulanic acid than the zone around the disc without the inhibitor. A quality control of the test was performed using *E. coli* ATCC 25922 (ESBL-negative) and *K*. *pneumoniae* ATCC 700603 (ESBL-positive).

### 4.5. Determination of ESBL Genotype

Genomic DNA was prepared from the tested strain cultured for 18 h in L-broth by using a commercial Genomic Mini Kit (A&A Biotechnology, Gdańsk, Poland) according to the manufacturer’s instructions. DNA suspended in Tris buffer (10 mM Tris, pH = 8) was stored at −20 °C.

A CTX-M group 1-specific pair of oligonucleotides, previously designed by Ensor et al. (2007), was used for amplification: 1,8F (F—forward) GCS^a^ ATG TGC AGC ACC AGT AA; 1R (reversed) ACA AAC CGT Y^a^GG TGA CGA TT. ^a^Oligonucleotides with degenerate bases S or Y were synthesized as an equal mixture of G and C and C and T, respectively [27]. The oligonucleotides were synthesized by Bionovo. PCR amplification was performed at a final volume of 50 μL, containing 1 μL of template DNA, 1 μL of each primer, and a 25 μL HotStarTaq^TM^ DNA polymerase Master Mix Kit (Qiagen, Hilden, Germany). PCR was performed in a thermocycler (Eppendorf, Juelich, Germany) according to the optimal conditions as follows: 1 cycle at 95 °C for 5 min, followed by 25 cycles of 95 °C for 30 s, 63 °C for 1 min, and 72 °C for 1 min, with a final elongation at 72 °C for 10 min. PCR products were submitted to 1% agarose gel electrophoresis in TAE buffer (40 mM Tris, pH 7.6; 20 mM C_2_H_4_O_2_, 1 mM EDTA) at 70 V. The amplicons were visualized using an ultraviolet transilluminator (GelDoc 2000, Bio-Rad, Hercules, CA, USA) after staining with ethidium bromide. The presence of a band of the 666 bp indicated the carriage of *bla*_CTX-M_ group 1 by the tested isolate. A 100–3000-bp DNA ladder (BLIRT DNA-GDAŃSK, Poland) was used as a molecular weight standard.

### 4.6. The LPS Extraction

The LPS was extracted from the bacterial mass of the *K*. *oxytoca* 0.062 strain obtained from 18 h aerated culture in nutrient broth containing 0.2% of Glc, which was killed by 90% phenol and rinsing twice with distilled water by centrifugation (5300× *g*, 1 h). The phenol–water extraction of LPS (65 °C, 30 min) was performed according to the procedure by Westpahl and Jann (1965), followed by LPS purification with 50% trichloroacetic acid, dialysis to water, and centrifugation (5300× *g*, 1 h) [43].

### 4.7. Degradation of LPS and Isolation of the Polysaccharide Fraction

An LPS sample (95 mg) of the *K. oxytoca* strain 0.062 was heated with aqueous 1.8% acetic acid at 100 °C for 3 h, and a lipid A precipitate was removed by centrifugation (11,000× *g*, 30 min). The carbohydrate-containing supernatant was concentrated and then fractionated by gel-permeation chromatography on a column (1.8 × 80 cm) of Sephadex G-50 fine (Pharmacia, Uppsala, Sweden) using 1% acetic acid as the eluent and monitoring with a differential refractometer (Knauer, Berlin, Germany). The yield of the polysaccharide fraction was 12% of the LPS portion subjected to hydrolysis.

### 4.8. Analytical Procedures

Sugar and methylation analyses were carried out as described in [44].

Monosaccharides were determined after conversion into alditol acetates or acetylated methyl glycosides and analyzed by GLC-MS using an Agilent Technologies 7890A gas chromatograph (USA), equipped with an HP-5MS capillary column (Agilent Technologies, 30 m × 0.25 mm), with helium as the carrier gas (flow rate of 1 mL min^−1^), and connected to a 5975C MSD detector (inert XL EI/CI, Agilent Technologies, Wilmington, DE, USA). The applied temperature program for the analysis of all sugar derivatives was as follows: 150 °C for 5 min, then 150 to 310 °C at 5 °C min^−1^, and the final temperature was maintained for 10 min.

The polysaccharide was hydrolyzed with 10 M HCl (80 °C, 30 min) and the methylated polysaccharide with 2 M CF_3_CO_2_H (100 °C, 4 h), reduced with NaBD_4_, and peracetylated with a 1:1 (*v*/*v*) Ac_2_O-pyridine mixture (85 °C, 30 min). The polysaccharide was subjected to methanolysis with 2 M HCl/MeOH (85 °C, 2 h) or 1 M HCl/MeOH (85 °C, 16 h), and the acetylated methyl glycosides were analyzed by GLC under the same conditions as above.

Methylation analysis was performed with methyl iodide in dimethyl sulfoxide in the presence of powdered sodium hydroxide according to the Ciucanu–Kerek procedure [45]. The partially methylated alditol acetates (PMAAs) were analyzed by GLC-MS. The absolute configuration of Man and Gal was determined by the GLC of acetylated (*S*)-(+)-2-octyl glycosides, as described previously [46]. In turn, the absolute configuration of GalA was determined based on the analysis of glycosylation effects on ^13^C resonances in the polysaccharide.

### 4.9. NMR Spectroscopy

For NMR spectroscopy, the polysaccharide sample (8 mg) was freeze-dried twice from a 99.95% D_2_O solution and then examined in 99.98% D_2_O. One and two-dimensional NMR spectra were recorded at 32 °C on a 500 MHz NMR Varian Unity Inova instrument (Varian Associates, Palo Alto, CA, USA) and chemical shifts were reported relative to acetone (δ _H_ 2.225, δ _C_ 31.45) as an external reference. Standard Varian software Vnmrj V. 4.2 rev. (Agilent Technologies, Santa Clara, CA, USA) was used to acquire and process the NMR data. The experiments including ^1^H,^1^H TOCSY, ^1^H,^1^H DQF-COSY, ^1^H,^1^H ROESY, ^1^H,^13^C HSQC, ^1^H,^13^C HSQC-TOCSY, ^1^H,^13^C H2BC, and ^1^H,^13^C HMBC were performed for signal assignments and the determination of the sugar sequence in the repeating unit. A mixing time of 100 and 200 ms was used in the TOCSY and ROESY experiments, respectively. The ^1^H,^13^C HSQC spectrum (band-selective gHSQCAD), measured without ^13^C decoupling, was used to determine the ^1^*J*_C1,H1_ coupling constants for the anomeric carbons.

### 4.10. SDS-PAGE Electrophoresis, Western Blotting, and Silver-Staining Procedures

The solution of each LPS (2 mg/mL) was mixed (1:1) with the sample buffer (2% SDS, 50 mM Tris/HCl (pH 6.8), 25% glycerol, 11% 2-mercaptoethanol, 0.1% bromophenol blue), boiled for 10 min, centrifuged, and loaded in stacking gel channels (6 µg/lane). Samples run on 12% SDS-polyacrylamide gels at 200 V in Tris-Glycine buffer. The separated LPSs were transferred to a nitrocellulose membrane (Whatman Schleicher & Schuel, Dassel, Germany) in buffer containing 25 mM Tris/HCl, 192 mM of glycine, and 20% methanol at 100 V per hour, and subjected to Western immunoblotting using LPS-specific anti-sera. Each membrane was incubated per night with *Proteus* antiserum diluted (1:100) in a dot blot 10% skimmed milk buffer (50mM of Tris/HCl pH 7.4, 200 mM of NaCl). Goat anti-rabbit-IgG antibodies conjugated with alkaline phosphatase (Jackson ImmunoResearch, West Grove, PA, USA) were used as secondary antibodies. The reactions were visualized by using the AP Conjugate Substrate Kit (Bio-Rad, Hercules, CA, USA). The 3-Color High Prestained Protein Marker (10–245 kDa) DNA GDAŃSK was used to indicate the range of cross-reaction. The blots were scanned by means of Canon Toolbox 4.9.

LPSs of *K*. *oxytoca* 0.062 and of *Proteus* spp. strains, cross-reacting in Western blotting, were separated by SDS-PAGE electrophoresis and directly stained using gel prestaining with 0.01% or 0.1% Alcian blue [in 40% (*v*/*v*) ethanol and 5% (*v*/*v*) acetic acid] at 37 °C for 2 h and at 20 °C for a night, followed by the silver staining method by Tsai and Frash (1982), with modifications [47,48]. After the prestaining step, the gel was incubated for 5 min in 0.7% sodium periodate in 40% ethanol and 5% acetic acid to generate carbonyl groups during the oxidation of LPS. After three 15 min washes in water, the gel was incubated for 10 min in a freshly prepared staining reagent (2 mL of cold ammonium hydroxide, 1 mL of 1N NaOH, 5 mL of 20% sliver nitrate/150 mL of water), followed by washing the gel with water. The LPSs were visualized by treating the gel with a developer (0.5 mL of 37% formaldehyde, 50 mg of citric acid/liter of water). The gel was scanned by means of Canon Toolbox 4.9.

## Figures and Tables

**Table 1:** The MIC values of antibiotics towards the tested *K*. *oxytoca* 0.062 strain and the negative control *E*. *coli* ATCC 25922. The data marked in purple refer to the resistant phenotype.

The Strain	Norfloxacin	Amoxicillin/ Clavulanic Acid	Ampicillin	Cephalothin	Cefuroxim	Trimethoprim/ Sulfametoxazole	Nitrofurantoin
*E*. *coli* ATCC 25922	0.5	4	4	4	2	1	1
*K*. *oxytoca* 0.062	1	16	>512	>512	>512	>512	16

**Table 2:** ^1^H and ^13^C NMR data of the polysaccharide antigens of *K. oxytoca* strain 0062.

Sugar Residue	Unit	H-1 C-1	H-2 C-2	H-3 C-3	H-4 C-4	H-5 C-5	H-6 C-6
→2)-α-D-Man*p*-(1→ ^1^*J*_C,H_ = 175 Hz; ^3^*J*_H1,H2_ = ~2 Hz	**A**	5.34 101.9	4.12 79.7	4.01 71.3	3.71 68.2	3.75 74.5	3.76; 3.88 62.2
→2)-α-D-Man*p*-(1→ ^1^*J*_C,H_ = 175 Hz; ^3^*J*_H1,H2_ = ~2 Hz	**B**	5.25 95.8	4.05 81.2	4.05 71.5	3.73 67.8	3.87 73.9	3.82; 3.92 62.0
→3,4)-α-D-Gal*p*A-(→ ^1^*J*_C,H_ = 174 Hz; ^3^*J*_H1,H2_ = ~3.5 Hz	**C**	5.22 102.1	4.08 69.2	4.18 78.2	4.64 79.1	4.58 72.1	– 175.1
→2)-α-D-Man*p*-(1→ ^1^*J*_C,H_ =174 Hz; ^3^*J*_H1,H2_ = ~2 Hz	**D**	5.15 101.2	4.28 78.2	3.87 70.9	3.72 68.1	3.77 74.5	3.82; 3.92 61.9
T-α-D-Man*p*3OMe-(1→ ^1^*J*_C,H_ = 174 Hz, ^3^*J*_H1,H2_ = ~2 Hz	**E’**	5.15 103.6	4.32 67.0	3.61 81.0	3.71 67.6	3.75 74.2	3.76; 61.9
T-α-D-Man*p*-(1→ ^1^*J*_C,H_ = 174 Hz; ^3^*J*_H1,H2_ = ~2 Hz	**E**	4.99 101.4	3.88 71.4	3.84 71.3	3.70 67.9	4.06 73.8	3.79; 3.90 62.1
→3)-β-D-Man*p*-(1→ ^1^*J*_C,H_ = 163 Hz; ^3^*J*_H1,H2_ = ~2 Hz	**F**	4.79 99.5	4.17 71.8	3.73 81.5	3.71 67.4	3.41 77.3	3.75; 3.93 62.2
→3)-β-D-Gal*p*-(1→ ^1^*J*_C,H_ = 161 Hz; ^3^*J*_H1,H2_ = 8 Hz	**G**	4.66 105.6	3.63 70.9	3.74 77.6	4.13 65.6	3.66 76.2	3.75;3.84 62.3

Chemical shifts of OMe group were δ_H_ 3.46, δ_C_ 57.6. The units marked in blue and black indicate the sugar residues of the neutral and acidic polysaccharide, respectively.

## Data Availability

Data is contained within the article and Supplementary Material.

## Supplementary Materials

The supporting information can be downloaded at: https://www.mdpi.com/article/10.3390/ijms26073177/s1.

## Abbreviations

The following abbreviations are used in this manuscript:

ATCC	American Type Culture Collection
CAZ	Ceftazidime
CDT	Combination Disk Test
CPS	Capsular Polysaccharide
CTX	Cefoxitin
D-GalNAc	*N*-acetyl-D-galactosamine
D-Gal	D-Galactose
D-GlcA	D-glucuronic acid
D-GlcNAc	*N*-acetyl-D-glucosamine
ESBL	Extended-Spectrum β-lactamases
EUCAST	European Committee on Antimicrobial Susceptibility Testing
D-GalA	D-Galacturonic Acid
Gal*p*	Galactopyranose
GLC-MS	Gas Chromatography-Mass Spectrometry
L-Lys	L-Lysine
LPS	Lipopolysaccharide
L-Thr	L-Threonine
L-Rha	L-Rhamnose
L-Ser	L-Serine
Man*p*	Mannopyranose
MDR	Multidrug Resistant
MIC	Minimal Inhibitory Concentration
NMR	Nuclear Magnetic Resonance
OPS	O-polysaccharide
PAGE	Polyacrylamide Gel Electrophoresis
PCR	Polymerase Chain Reaction
*R*-Cet-L-Lys	N^ε^-[(*R*)-1-Carboxyethyl]-L-lysine
Rha	Rhamnose
(*S*)-Lac	(*S*)-Lactic acid [(*S*)-2-hydroxypropanoic acid]
