# Supplementary material for: The First Report on the Structure of Polysaccharide Surface Antigens of the Clinical Klebsiella oxytoca 0.062 Strain and the Contribution in the Serological Cross-Reactions
Source: Int J Mol Sci. 2025 Mar 29;26(7):3177. doi: 10.3390/ijms26073177 (PMC11989651; doi:10.3390/ijms26073177)
Supplement: Supplementary file 1 [file ijms-26-03177-s001.zip › ijms-3524093-supplementary.pdf]

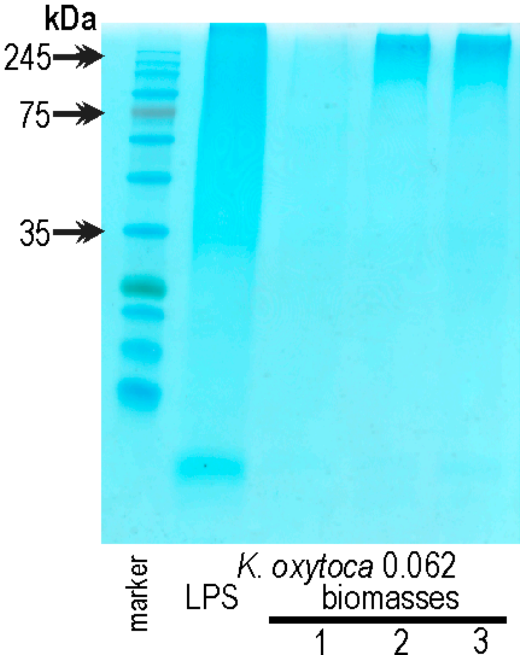

Figure S1: SDS-PAGE and Alcian blue staining patterns of the LPS from *K. oxytoca* 0.062 strain and its biomasses preparations: remained after CPS extraction (1); untreated with proteinase K (2); proteinase K treated (3). The gel was incubated in 0.1% Alcian blue in 40% (*v*/*v*) ethanol and 5% (*v*/v) acetic acid at 37 ° C for 2 hours and at room temperature for 18 hours.
